# Effect of pH on the Structure, Functional Properties and Rheological Properties of Collagen from Greenfin Horse-Faced Filefish (*Thamnaconus septentrionalis*) Skin

**DOI:** 10.3390/md22010045

**Published:** 2024-01-13

**Authors:** Kunyuan Wu, Yushuang Li, Junde Chen

**Affiliations:** Technical Innovation Center for Utilization of Marine Biological Resources, Third Institute of Oceanography, Ministry of Natural Resources, Xiamen 361005, China; 18900298990@163.com (K.W.); liyushuang@tio.org.cn (Y.L.)

**Keywords:** collagen, pH, secondary structure, functional properties, rheological properties

## Abstract

Collagen is an important biopolymer widely used in food, cosmetics and biomedical applications. Understanding the effect of pH on the structure and properties of collagen is beneficial for its further processing and exploitation. In this study, greenfin horse-faced filefish skin collagen (GHSC) was prepared and identified as a type I collagen. We systematically investigated the effect of pH on the structural, functional and rheological properties of GHSC. Scanning electron microscopy showed that the collagen morphology changed from an ordered stacked sheet structure to a rough silk-like structure as pH increased. Gaussian-fitted Fourier infrared spectroscopy results of the collagen revealed that it unfolded with increasing pH. Moreover, the ordered structure was reduced, and random coils became the dominant conformation. Its β-sheet and random coil contents increased from 18.43 ± 0.08 and 33.62 ± 0.17 to 19.72 ± 0.02 and 39.53 ± 1.03%, respectively, with increasing pH. α-helices and β-turns decreased from 35.00 ± 0.26 and 12.95 ± 0.01 to 29.39 ± 0.92 and 11.36 ± 0.10%, respectively. The increase in β-sheets and random coils allowed the pI-treated collagen to exhibit maximum water contact angle. The emulsification and foaming properties decreased and then increased with increasing pH in a V-shape. The increased net surface charge and β-sheets in collagen benefited its emulsification and foaming properties. The rheological results showed that the protoprotein exhibited shear-thinning properties in all pH ranges. The collagen solutions showed liquid-like behaviour in low-pH (2, 4) solutions and solid-like behaviour in high-pH (6, 7.83 and 10) solutions. Moreover, the frequency-dependent properties of the storage modulus (G′) and loss modulus (G″) of the collagen solutions weakened with increasing pH. Collagen has considerable frequency-dependent properties of G′ and G″ at low pH (2, 4). Thus, the importance of collagen raw material preparation for subsequent processing was emphasised, which may provide new insights into applying collagen-based materials in food, biomaterials and tissue engineering.

## 1. Introduction

Collagen is a natural biopolymer found in large quantities in the extracellular matrix of animal tissues, and it has been widely used in the food, cosmetic and pharmaceutical industries [1,2]. The collagen molecule consists of three α-chains with a unique (Gly-X-Y)n amino acid sequence pattern, resulting in rod-like triple helices of collagen. These protofibrils crosslink to form a molecular scaffold that provides mechanical strength and structural integrity to connective tissues [3]. The X amino acid remnants are typically proline or hydroxyproline, and Y represents any amino acid in (Gly-X-Y)n [4]. Gly is hidden in the triple-helix structure, but residues of X and Y are fully and partially exposed to solvent water, respectively, with basic and acidic residues occupying approximately 15–20% of the triple-helix structural domain of collagen [5]. Collagen is used as a scaffold biomaterial in tissue engineering and regenerative medicine owing to its natural properties, including good biocompatibility, haemostatic properties and weak antigenicity [6].

Collagen extracted from porcine and bovine presents the risk of zoonotic disease transmission and religious restrictions [7]. Marine collagen is an alternative and safer source of collagen that is accepted by Muslim- and Hindu-based countries. Compared with mammalian collagen, marine collagen has the advantages of high extraction yield, easier preparation and high functional properties. The extraction yields of *Megalonibea fusca* swim bladders collagen (33.38%) [8] and double-spotted pufferfish (*Takifugu bimaculatus*) skin collagen (49.83 ± 1.85%) [9] were higher than those of cattle tendon collagen (2.4–6.2%) [10], pork skin collagen (11.38% ± 0.66%) [11] and sheep slaughter by-product collagen (12.05%) and lamb slaughter by-product collagen (18.0%) [12]. The emulsifying activity index of collagen of red stingray (*Dasyatis akajei*) skin collagen (61.06–117.11 m^2^/g) [13] was superior to that of bactrian camel (*Camelus bactrianus*) hoof collagen [14] and rabbit meat collagen (44.7 m^2^/g), rabbit skin collagen (46.6 m^2^/g), rabbit ear collagen (48.2 m^2^/g) [15] and lamb slaughter by-product collagen (59.1 m^2^/g) [12]. The emulsifying stability index of double-spotted pufferfish skin collagen (45.23–13.68 min) [9] was higher than that of rabbit meat collagen (9.8 min), rabbit skin collagen (9.8 min), and rabbit ear collagen (9.8 min) [15]. Because of the high demand and unique physicochemical properties of marine collagen, scientific and industrial interest in marine collagen is growing, and the marine collagen market value has been reported to reach USD 1055.2 million by 2026 [2]. This is promising for the use of processed waste, such as fish skins, bones and swim bladders, which can be employed to extract collagen for high-value utilisation. Recently, marine collagen has been prepared and characterised by many researchers. Chen et al. (2021) [4] extracted the scale collagen of the Lizardfish (*Synodus macrops*), Mo et al. (2023) [8] isolated pepsin and acid-soluble collagen from the swim bladders of *Megalonibea fusca*, and Devita et al. (2021) [16] isolated type I collagen from bigeye tuna (*Thunnus obesus*) skin via the acid–enzymatic method. The greenfin horse-faced filefish (*Thamnaconus septentrionalis*), also known as the “skinned fish” in China, is a species of tetraodontiform fish widely distributed in the Indo–West Pacific Ocean [17,18]. The filefish is an important economic fish species in China, and according to the China Fisheries Statistical Yearbook, the marine catch of filefish reached 122,258 tonnes in 2023. The greenfin horse-face filefish is an important commercial species in China, Korea and Japan, owing to its high protein content and pleasant taste [18]. However, “skinned fish” must be removed before eating due to its rough skin characteristics [18], which results in a large amount of skin being wasted as trash. Fish skin has been reported to be a rich source of collagen, with collagen isolated from it accounting for 70–80% of the weight of dried skin [19]. However, there are few reports on collagen from the skin of the greenfin horse-faced filefish.

pH is important for collagen processing and application. It is well known that pH plays a key role in the strength of electrostatic interactions because it determines the charge density of proteins [20]. This interaction can result in soluble complexes, affecting the protein’s physicochemical and functional properties. Notably, collagen is an amphoteric electrolyte with a pH-responsive function [21]. Each collagen peptide chain has several acidic or basic side groups, all of which can accept or provide protons. The effect of pH on collagen is primarily related to the isoelectric point (pI) of the protein. pH affects the solubility of collagen molecules, the interaction between collagen molecules and the molecular conformation of collagen [22]. As the pH changes, the dissociable groups become either neutral or charged within a given pH range. Therefore, it would be beneficial to study the effect of pH on the structure, functional properties and rheological behaviour of collagen, which could facilitate its utilisation in food, cosmetics and medicine. However, the effects of pH on the structural and functional properties and the rheological behaviour of greenfin horse-faced filefish skin collagen (GHSC) have not yet been reported.

For this reason, the aim of this study was to isolate collagen from the skin of greenfin horse-faced filefish and investigate the effect of pH induction on the structure and properties of GHSC. 

## 2. Results and Discussion

### 2.1. Yield

The yield of GHSC extracted from greenfin horse-faced filefish skin was 46.1 ± 2.1% (based on a dry weight basis), which was higher than that of bigeye tuna (*Thunnus obesus*) skin collagen (13.5 ± 0.6%) [23], cattle tendon collagen (2.4–6.2%) [10] and pork skin collagen (11.38 ± 0.66%) [11]. This difference in collagen extraction yield can be attributed to various factors such as different methods of raw material preparation, different extraction methods, and different raw materials (species).

### 2.2. Sodium Dodecyl Sulphate–Polyacrylamide Gel Electrophoresis (SDS–PAGE) of GHSC

**Figure 1:**
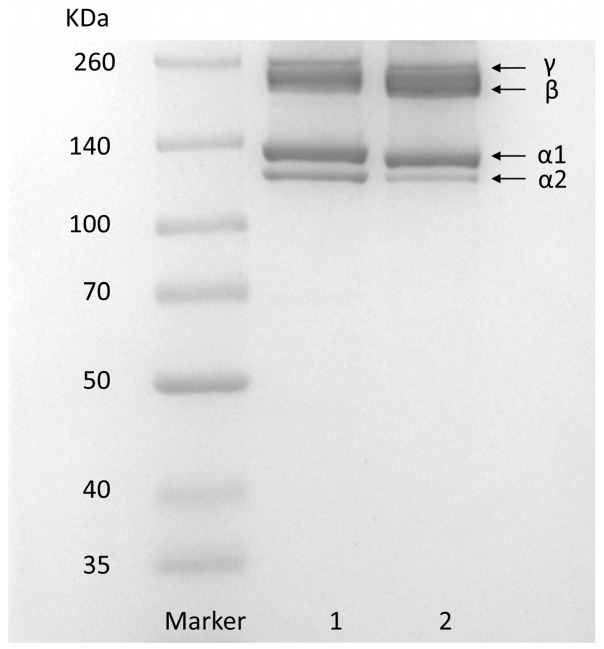
SDS-PAGE pattern. (1) rat tail collagen; (2) GHSC.

The SDS–PAGE of GHSC is shown in Figure 1. Type I rat tail collagen was used as the standard. GHSC has a similar protein pattern to rat tail collagen, which primarily consists of two different α-chains, α1 and α2. In addition, high-molecular-weight dimer β-chains and trimer γ-chains were observed, and the molecular weights of α1-chains, α2-chains, β-chains and γ-chains of GHSC were found to be 125, 132, 227 and 252 kDa, respectively, using the Quantity one 4.6.0 software (Bio-Rad Laboratories, Hercules, CA, USA). Analysis using ImageJ 1.8.0 software (National Institute of Mental Health, Bethesda, MD, USA) revealed that the grey scale ratio of α1-chains to α1-chains was close to 2:1, which is consistent with the [α1]2α2 structure of typical type I collagen [7]. This suggests that the prepared GHSC is a typical type I collagen. Additionally, the purity of GHSC was estimated by SDS-PAGE using ImageJ [24]. The quantification of stained protein bands showed that α1, α2, β, and γ subunits contributed to 95.87 ± 3.00% of the total GHSC, indicating that GHSC had high electrophoretic purity.

### 2.3. Zeta Potential

**Figure 2:**
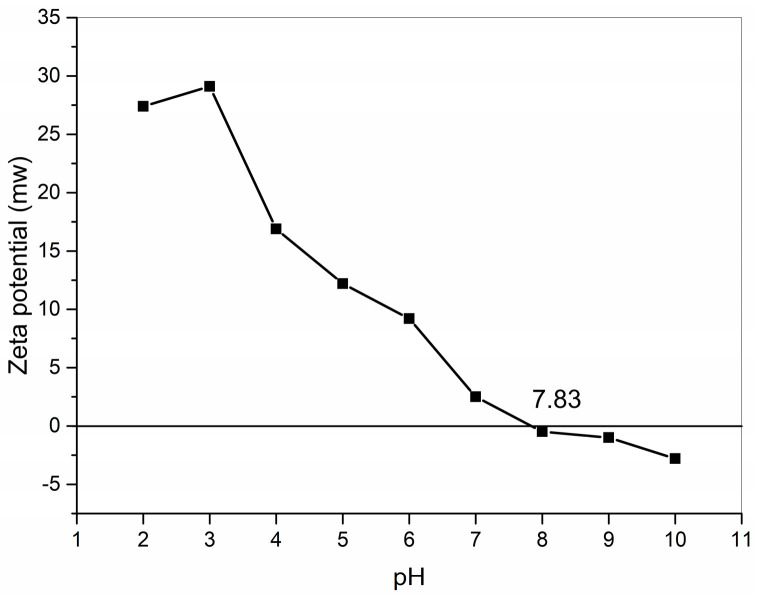
Zeta potential of GHSC.

The zeta potential is a physicochemical property that is one of the most useful parameters for understanding charge interactions [25]. Collagen is an amphoteric electrolyte with a pH-responsive function [21], and measuring the collagen pI is important for further applications. Each peptide chain of collagen has many acidic or basic side groups, and each peptide chain has α-carboxylic and α-amino groups at the ends, which can dissociate to produce a positive or negative charge in a specific pH range. The pI of GHSC was obtained from zeta potential tests (Figure 2). For GHSC with a pI of 7.83, the zeta potential was zero, the net charge of the collagen surface was zero, the electrostatic repulsion between collagen chains was reduced, and this GHSC had the lowest solubility [26]. When the pH is lower than pI, the α-carboxylic or acidic side groups dissociate by binding with H^+^, making the collagen surface positively charged with an electropositive zeta potential. And when the pH is lower than pI, the α-amino or basic side groups dissociates by binding with OH^–^, making the collagen surface negatively charged and an electronegativity display zeta potential. This makes the collagen solution exhibit pH dependence.

### 2.4. Morphology Characterisation

**Figure 3:**
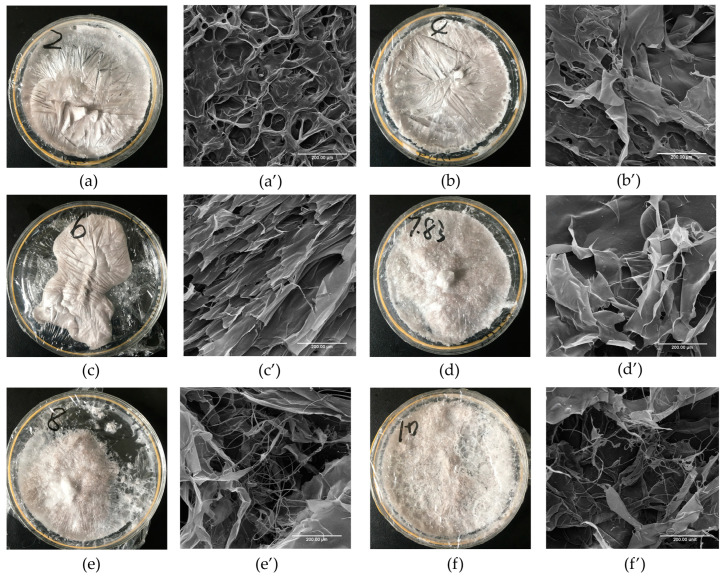
Morphology Characterisation of pH-treated GHSC. (**a**–**f**): apparent morphology of collagen treated with pH 2, 4, 6, 7.83, 8 and 10, respectively; (**a′**–**f′**): microscopic morphology of collagen treated with pH 2, 4, 6, 7.83, 8 and 10, respectively.

The apparent structure and microscopic morphology of GHSC are shown in Figure 3. Figure 3a–f show that the collagen has different morphologies with pH. The lyophilised GHSC samples resembled sponges with a loose, homogeneous and porous structure. The structure of the lyophilised GHSC gradually changed from a distinctly textured and compact structure at the beginning to a rough and loose surface as the pH increased. As shown in scanning electron microscopy (SEM) images (Figure 3a′–f′), the microscopic morphology of the low pH-treated GHSC included more aggregated structures. At pH 2, GHSC had a porous structure with a tightly packed but disordered stack, and it was difficult to observe its sheet-like structure, which could be due to the extremely acidic conditions. With increasing pH, GHSC was in the form of large, orderly, stacked sheet-like structures (pH 6). As the pH was further increased (pH 8–10), the SEM image showed that the sheet-like structure became smaller and had more loose silk-like structures. This could be because pH led to a change in internal interactions of the collagen, which changed the collagen structure. Previous literature reported that the fibrous and sheet membrane structure of collagen facilitates cell growth, wound healing and new tissue formation [8]. Therefore, it could be used in practical biomedical applications, such as wound management, biofilms and drug delivery by modifying the morphology upon adjusting the pH.

### 2.5. Secondary Structure

**Figure 4:**
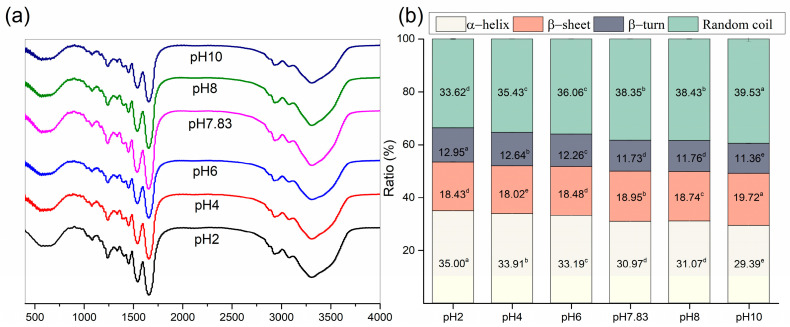
Spectral information of pH-treated GHSC. (**a**) FTIR spectrum; (**b**) secondary conformation content. Different letters (a–e) indicate significant differences between samples.

Fourier transform infrared (FTIR) of pH-treated collagen showed five typical characteristic peaks of collagen: the characteristic bands of amide A, amide B, amide I, amide II and amide III (Figure 4a) [7]. The amide I band (1600–1700 cm^−1^) from infrared absorption is present mainly due to the stretching vibration of the carbonyl group in the polypeptide chain and represents the secondary structure of proteins [27]. Figure 4a shows the amide I bands of pH-treated collagen with peak positions at 1657, 1656, 1655, 1653, 1653 and 1655 cm^−1^ at pH 2–10. The reason for the slight shift in the amide I band may be related to pH induction. To further understand the effect of pH on collagen structure, we performed Gaussian fitting of the amide I band and analysed the changes in secondary structure of pH-treated collagen. There are four main collagen secondary structure types: α-helices, β-sheets, β-turns and random coils. As mentioned in previous literature, the range of α-helices is generally from 1646 to 1664 cm^−1^, the range of β-turns is generally from 1664 to 1681 cm^−1^, the range of β-sheets is generally from 1615 to 1637 cm^−1^ (low band) and from 1682 to 1700 cm^−1^ (high band), and the range of random coils is generally from 1637 to 1645 cm^−1^ [28]. α-helices are more tightly ordered secondary structures than β-sheets. Both α-helices and β-sheets are highly stable and contain more hydrogen bonds, making the protein structure regular and compact. β-turns and random coils are typically found in disordered secondary structures with poorer stability, making the protein molecular structure looser and more flexible [27,28].

**Figure 5:**
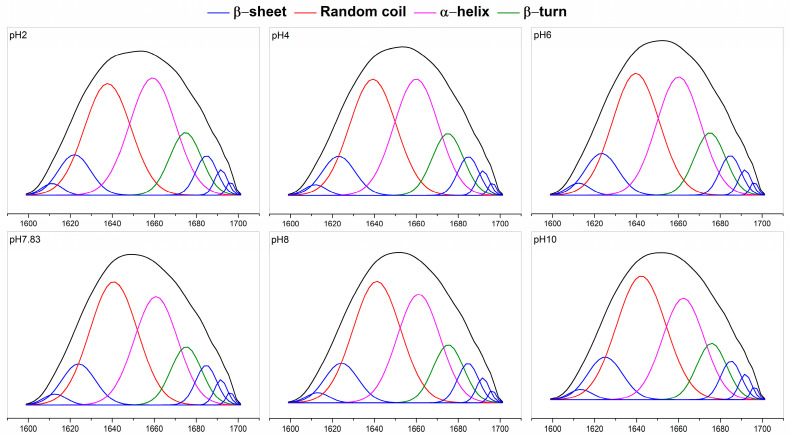
Gauss fitting curves of pH-treated GHSC obtained from the 1700–1600 cm^−1^ spectrum.

The fitted curves are shown in Figure 5, and eight sub-peaks were obtained after Gaussian fitting of the amide I band. The corresponding secondary structures and their contents are shown in Figure 4b. The contents of α-helices and β-turns decreased from 35.00 ± 0.26% and 12.95 ± 0.01% to 29.39 ± 0.92% and 11.36 ± 0.10%, respectively, and the β-sheets and random coils increased from 18.43 ± 0.08% and 33.62 ± 0.17% to 19.72 ± 0.02% and 39.53 ± 1.03%, respectively, upon increased pH from 2 to 10. For collagen treated at pH 2, α-helices and random coils were the major components of found in the secondary structure. However, with increasing pH, the contents of α-helices and β-turns decreased, and the contents of β-sheets and random coils increased. Random coils became the dominant conformation of the collagen secondary structure. Meanwhile, a sudden increase in the content of β-sheets to 18.95 ± 0.01% was observed at pH 7.83, which might be related to the reduced collagen solubility at the pI, resulting in more exposed hydrophobic groups. Xiang et al. (2018) [29] reported a positive correlation between the number of β-sheets and surface hydrophobicity. These results suggested that pH could change the secondary structure of collagen. As pH increased, the collagen unfolded, and its internally ordered structure was reduced.

### 2.6. Water Contact Angle (WCA)

**Figure 6:**
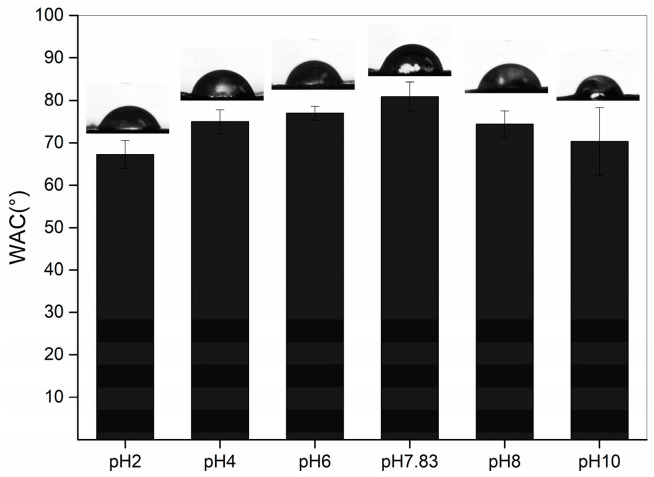
Water contact angles of pH-treated GHSC.

The WCA indicates the hydrophilicity of the collagen surface, and WCA increases with increasing hydrophobicity. The WCA of the pH-treated collagen is shown in Figure 6. With different pH treatments, it showed a tendency to increase and then decrease, with a maximum value of 80.85 ± 3.47° at the pI. As the pH was increased from 2 to 7.83, the WCA of collagen increased from 67.21 ± 3.33 to 80.85 ± 3.47°. The hydrophilicity of the collagen surface weakened, probably due to the increase in pH, which altered the intermolecular interactions within the collagen, exposing more hydrophobic groups. This is consistent with the results of Xiang et al. (2018) [29], who concluded that hydrophobic sites can be largely exposed in proteins with a looser molecular structure (low α-helix content but high in β-sheets and random coils). This is consistent with the results obtained for the secondary structure, with the site at pH 7.83 possessing the highest content of β-sheets, a random coil structure and the lowest content of α-helices. However, as the pH was further increased from 7.83 to 10, we observed a decrease in WCA, with the lowest WCA of 70.30 ± 7.93° at pH 10. This could be attributed to the loose and rough morphology (Figure 3f), leading to an increase in hydrophilicity of the collagen surface despite the continued decrease in α-helices and a further increase in β-sheets and the random coil structure. This structure also resulted in a broader distribution of the WAC for pH 10-treated collagen than at other pHs.

### 2.7. Functional Properties

#### 2.7.1. Emulsifying Activity Index (EAI) and Emulsifying Stability Index (ESI)

**Figure 7:**
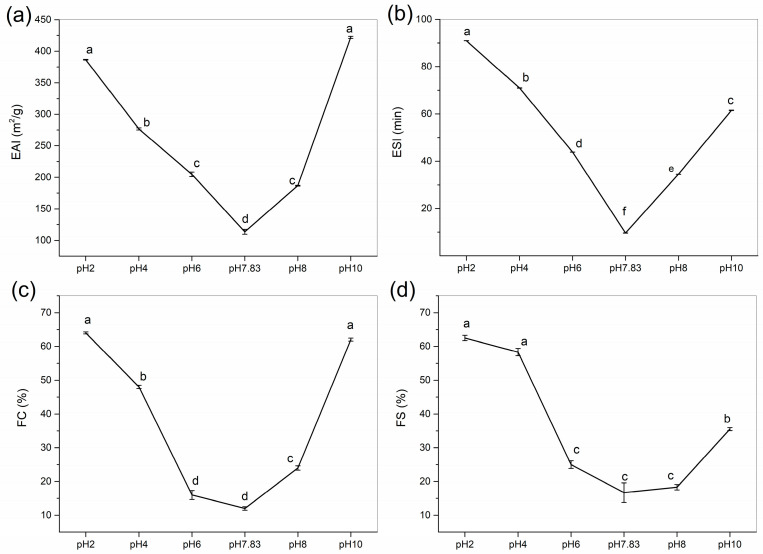
Functional properties of GHSC. (**a**) EAI; (**b**) ESI; (**c**) FC; (**d**) FS. Different letters (a–f) indicate significant differences between samples.

Emulsifying ability measures the effectiveness of proteins as emulsifiers and characterises the ability of protein emulsions to adsorb rapidly at the oil–water interface [30]. The emulsifiability and emulsion stability of collagen were determined at pH 2–10, and the effects of pH treatment on the EAI and ESI of collagen are shown in Figure 7a,b. We found that to use GHSC in applications that require high emulsifiability and emulsion stability, we should use a pH that is not near the pI. Similar results were found by Li et al. (2022) [28], where EAI and ESI were V-shaped with increasing pH, with the lowest values at the pI.

The EAI of collagen decreased from 386.61 ± 0.55 to 113.56 ± 3.94 m^2^/g as the pH was increased from 2 to 7.83, and then it increased to 421.89 ± 1.59 m^2^/g as the pH was increased from 7.83 to 10. The EAI for GHSC was found to be higher than the previously reported rabbit meat collagen (44.7 m^2^/g), rabbit skin collagen (46.6 m^2^/g), rabbit ear collagen (48.2 m^2^/g) [15], chicken feet collagen (71.4 m^2^/g) [31], and lamb slaughter by-product collagen (59.1 m^2^/g) [12]. Ngui et al. (2021) [32] reported that solubility provides proteins with a better ability to diffuse into the oil–water interface, resulting in lower interfacial tension and improved protein emulsification properties. When the pH is less than the pI, the net collagen surface charge decreases with increasing pH, and collagen solubility reduces. Even though secondary structure analyses showed a gradual unfolding of collagen and an increase in β-sheets and flexible random coil conformations, solubility still has the greatest influence on the emulsifying properties of collagen. In a solution at a pI of 7.83, the emulsion shows uncharged surface droplets, and the proteins tend to aggregate and precipitate. At this point, the proteins have low solubility, making absorption at the oil–water interface insufficient to form a homogeneous emulsion, resulting in the lowest EAI. When the pH is higher than the pI, increased solubility and collagen defolding might be responsible for the increase in EAI. The net surface charge of collagen increases with pH, allowing dissolved proteins rapid adsorbtion at the oil–water interface [32]. This promotes the formation of a film around the oil droplets and contributes to emulsion formation. Moreover, with increasing pH, collagen has an extended secondary structure that produces more flexible and random coil conformations, which facilitates faster adsorption of molecules at the oil–water interface and enhances the emulsifying activity of proteins [33,34,35].

Emulsion stability is the ability of a protein to maintain an emulsion. The trend of the ESI in this collagen was consistent with the EAI. As shown in Figure 7b, the ESI decreased and then increased with increasing pH. The ESI decreased from 90.93 ± 0.02 min to 9.63 ± 0.04 min and then increased to 61.48 ± 0.10 min. This is consistent with the result of Lu et al. (2021) [34], who reported that the larger the absolute value of the zeta potential, the more adequate the adsorption of the sample on the surface of oil droplets, making the electrostatic repulsive forces between the droplets strong enough to maintain emulsion stability. At pH 7.83 (pI), the electrostatic charge on the collagen surface was almost zero, and the repulsive force between the droplets made it difficult to maintain emulsion stability, which resulted in the minimum ESI at the pI for collagen, with a V-shaped ESI curve. Therefore, by controlling the pH of the collagen solution system, collagen can be used as an emulsifier and potentially as a delivery material in food and pharmaceutical applications [36,37].

#### 2.7.2. Foaming Capacity (FC) and Foaming Stability (FS)

Figure 7c,d show the effect of pH on FC and FS of GHSC. The FC and FS of collagen first decreased and then increased with increasing pH. This trend was identical to those of EAI and ESI, which exhibited the lowest values when the pH was equal to the pI. Figure 7c,d show that FC decreased from 64.0 ± 0.3 to 12.0 ± 0.5% when the pH was increased from 2 to 7.83 and then increased to 62.0 ± 0.45% when the pH was increased to 10. The results for FC in this study were higher than sheep slaughter by-product collagen (9.58%) [12], rabbit meat collagen (9.2–11.7%) and rabbit skin collagen (4.2–9.2%) [15]. The FS of GHSC ranged from 62.5 ± 0.81 to 16.7 ± 2.9% for pH 2–10, with a minimum at pI, and the FS result of GHSC was higher than that of chicken feet collagen (11.7%) [31]. These results indicate that solubility has the main influence on the foaming properties of collagen at pHs below the pI. Moreover, that low solubility limits the ability of the protein to form foam at the water–air interface [38]. Although protein unfolding has been reported to promote high protein foaming and foam stability, when the pH is above pI, the combination of the solubility and secondary structure affects collagen’s foaming properties [39]. The increased collagen solubility leads to faster diffusion of collagen into the air–water interface, thereby increasing foam formation. Moreover, increasing the pH leads to increased random coil conformations in the secondary structure, increasing its flexibility. This enhances the adsorption of the protein at the air–water interface, improving the foaming ability of the protein [38,40]. Therefore, by controlling the pH during processing, collagen could be a good candidate for products that require foaming properties, such as food drug delivery foams for consumers with special needs [41,42].

### 2.8. Rheological Properties

**Figure 8:**
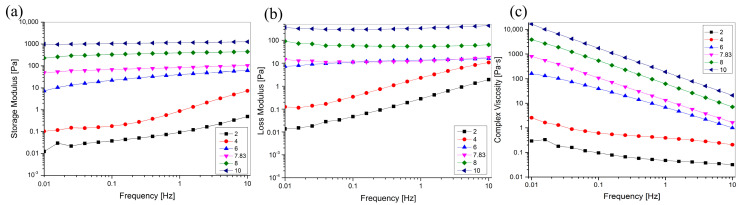
Effect of pH on the rheological behavior of GHSC solution. (**a**) The storage modulus G′; (**b**) The loss modulus G″; (**c**) The complex viscosity η*.

Rheological properties are necessary to understand to perform suitable mechanical and process calculations, and understanding the effect of pH on the rheological properties of collagen helps manufacturers control or manipulate the physical properties during the development of collagen-based materials to produce stable products. To investigate the effect of pH on viscoelasticity, we carried out oscillatory rheological frequency sweeps of pH-treated collagen in the range of 0.01–100 Hz, and the results are shown in Figure 8. Figure 8c shows that the complex viscosity increased with increasing pH, indicating that the viscosity of the collagen solution could be controlled by adjusting solution pH. G′ and G″ increased with increasing pH in the sweep frequency range of 0.01–10 Hz, indicating that pH can improve the viscoelasticity of the collagen solution. Moreover, when the pH (8, 10) was greater than pI, G′ was higher than G″ in the scanning frequency range of 0.01–10 Hz. This suggests that elasticity is preferred to viscosity and that elastic behaviour is dominant in solution systems, with solutions exhibiting solid-like behaviour. When the pH (2, 4, 6) is lower than the pI, the dominant role of elastic behaviour gradually decreases, viscous behaviour becomes dominant, and the solution exhibits liquid-like behaviour. The unique viscoelastic properties of collagen facilitate its use in tissue engineering and drug delivery applications, such as injectable biomaterials and wound dressings [43,44,45].

The complex viscosity of the pH-treated collagen solutions decreased with increasing sweep frequency (Figure 8c), suggesting that all pH-treated collagen solutions exhibited non-Newtonian shear thinning behaviour, which is generally suitable for practical applications, such as those requiring syringeability and easy spreadability [46]. With an increase in pH from 2 to 10, the G′ and G″ of the collagen solution at 0.01 Hz increased from 0.0124 and 982 to 0.493 and 1260 Pa at 10 Hz (Figure 8a,b). This indicated that the change in frequency affected the collagen solution more significantly at low pH than at high pH. This is also evident in Figure 8a,b, where it is shown that G′ and G″ of the collagen at pH 2 and 4 significantly increased with increasing sweep frequency. The smoother G′ and G″ at pH 6–10 relative to pH 2–4 suggested that the G′ and G″ of the collagen solution at low pH (pH 2–4) showed a stronger frequency dependency. They were less susceptible to frequency changes at high pH (pH 6–10). This resulted in a relatively stable viscoelasticity with increasing frequency (0.01–10 Hz).

## 3. Materials and Methods

### 3.1. Materials

The greenfin horse-faced filefish (*T. septentrionalis*) skin used as the raw material was purchased from an aquatic product processing plant in Zhangzhou, China and was kept at a low temperature during transport and stored at −20 °C. Protein markers (26634) were purchased from Thermo Fisher Baltic Sciences (Vilnius, Lithuania). All chemical reagents used in this study were of analytical grade.

### 3.2. Preparation of GHSC

The preparation method was as described by Chen et al. (2016) [7] with slight modifications. The greenfin horse-faced filefish skin was first thawed at 4 °C and cleaned with distilled water. The remaining meat and fat on the skin were removed, and the processed skin was cut into small pieces. The skin pieces were immersed in 0.5 M acetic acid and stirred for 24 h before centrifugation (Avanti J-26 XP, Beckman Coulter, Inc., Brea, CA, USA) at 9000 rpm for 30 min. The supernatant was collected, stirred with sodium chloride at a ratio of 1:25 (*w*/*v*) for 30 min, and then centrifuged at 9000 rpm for 30 min to collect the precipitate. It was then redissolved in 0.5 M acetic acid at a ratio of 1:9 (*w*/*v*). The supernatant obtained was dialysed in a dialysis bag with 0.1 M acetic acid for 24 h and then with deionised water for 48 h. The dialysed supernatant was lyophilised by freeze-drying (Telstar, lyoobeta-25, Barcelona, Spain).

### 3.3. Yield of GHSC

The yield of GHSC extraction was calculated using the following equation:

(1) $$Yield\;(\%)=\frac{m_{1}}{m_{2}}\times 100,$$

where *m*_1_ is the weight of lyophilized collagen and *m*_2_ is that of initial dry fish skin.

### 3.4. SDS–PAGE

The SDS–PAGE of the sample was determined according to Laemmli’s method (1970) [47], with slight modifications. The lyophilised samples were dissolved in ultrapure water at 0.1% (*w*/*v*). The resulting solution was mixed with a buffer (0.025 M Tris-HCl, pH 8.3, including 0.192 M glycine and 0.1% *w*/*w* SDS) at a ratio of 1:4 and then denatured at 100 °C for 3 min. SDS–PAGE of the samples was determined on a mini protein vertical slab electrophoresis system (Bio-Rad Laboratories, Hercules, CA, USA). Then, 6 μL of sample solution and marker (26634) was loaded on polyacrylamide gels consisting of 8% separating gel and 3% stacking gel. Electrophoresis was performed at a constant voltage of 110 V until the end of the separation. The gels were removed, fixed, stained and destained separately for 30 min.

### 3.5. Zeta Potential

Lyophilised samples were dissolved in a 0.1 M acetic acid to a final concentration of 0.002% (*w*/*v*). Then, 1.0 M HNO_3_ and 1.0 M KOH were used to adjust the pH of the sample solutions to 2, 3, 4, 5, 6, 7, 8, 9 and 10 [26,48,49,50]. The zeta potential of the samples was determined using a zeta potential analyser (Zetasizer Nano ZS90, Malvern Instr., Melvin, UK).

### 3.6. Morphological Characteristics

The morphologies of the samples at different pHs were observed using SEM (S-4800, HITACHI, Tokyo, Japan). Samples were adhered to the SEM stage with conductive tape and sputter-plated with gold. The surface morphology was then observed at an accelerating voltage of 15 kV, and SEM images were obtained at a magnification of 400×.

### 3.7. FTIR Spectroscopy

FTIR spectroscopy of the samples was performed according to Li et al. (2022) [28], with slight modifications. First, 1 mg of the sample was mixed with 100 mg of KBr at a ratio of 1:100. Then, the components were ground together under IR light drying conditions until they were free of particles, forming translucent sample flakes. FTIR spectra were recorded using a spectrometer (VERTEX 70, Bruker, Ettlingen, Germany) in the range of 400–4000 cm^−1^ with a single scan resolution of 4 cm^−1^. The amide I band (1700–1600 cm^−1^) in the FTIR spectrum of the samples was examined by second-order derivative analysis (OMNIC 8.2 software, Thermo Nicolet, Madison, WI, USA), followed by Gaussian curve fitting to obtain the fitted curves (PeakFit v4.12 software, SPSS Inc. Chicago, IL, USA).

### 3.8. WCA

The WCA was measured according to the method of Deng et al. (2018) [51], with slight modifications. The samples were fixed on glass slides. Then, a drop of distilled water (2 μL) was placed on the surfaces, and the WCA was measured using a contact angle tester (DSA 100, Kruse, Germany). The droplets were equilibrated for 3 s before the measurement. The static WCA of collagen was measured using the SCA 20 supplied with the instrument.

### 3.9. Functional Properties

#### 3.9.1. EAI and ESI

EAI and ESI were determined using the method of Ogunwolu et al. (2009) [52], with slight modifications. EAI and ESI of the sample were obtained using a homogeniser (JS25, Yangzhou Junrui Electromechanical Equipment Manufacturing Factory, Yangzhou, China). First, 15 mL of a 5 mg/mL sample solution and 5 mL of sunflower oil were homogenised using a homogeniser for 60 s at 22,000 rpm. The emulsion (50 μL) was withdrawn from the bottom first. Then, after 10 min, the emulsion was withdrawn and diluted 100-fold with SDS (0.1%, *w*/*v*). The absorbance of the solution was recorded at 500 nm using a UV spectrophotometer (UV-2550 Spectrophotometer, Shimadzu, Japan), and we performed three parallel tests. EAI and ESI were calculated as follows:

(2) $$EAI\;(m^{2}/g)=\frac{2\times 2.303\times A}{0.25\times Protein\;Concentration},$$

(3) $$ESI\;(min)=\frac{A_{10}\times \Delta t}{\Delta A},$$

where *A* is the absorbance at 0 min, *A*_10_ is the absorbance at 10 min, ∆*A* is *A*_0_ − *A*_10_, and ∆*t* is 10 min.

#### 3.9.2. FC and FS

FC and FS were determined according to Deng et al. (2019) [53], with slight modifications. First, 50 mL of a 5 mg/mL sample solution was homogenised at 16,000 rpm for 2 min using a JS25 homogeniser (Yangzhou Junrui Electromechanical Equipment Manufacturing Factory, Yangzhou, China). The volume of foam was then recorded at 0 min and 3 h. The equations for FC and FS were calculated as follows:

(4) $$FC\;(\%)=\frac{V_{2}-V_{1}}{V_{1}}\times 100,$$

(5) $$FS\;(\%)=\frac{V_{2}-V_{3}}{V_{2}-V_{1}}\times 100,$$

where *V*_1_ is the volume before whipping, *V*_2_ is the volume after whipping, and *V*_3_ is the volume after 3 h.

### 3.10. Rheological Properties

The rheological properties of the sample solutions were measured using a rotational rheometer (MCR 302, Anton Paar, Graz, Austria) with stainless steel cone/plate geometry (cone angle of 0.5°, cone diameter of 60 mm) and a gap of 57 µm. The G′, G″, and η* values were recorded as a function of frequency. All samples were tested at 25 °C with dynamic frequency sweeps from 0.01 to 10 Hz.

### 3.11. Statistical Analysis

All experiments were performed in triplicate, and the results are expressed as means ± standard deviation (SD). All data were analysed by ANOVA using SPSS Version 17.0 software (IBM SPSS Statistics, Ehningen, Germany), and *p* < 0.05 was used to indicate significant deviation.

## 4. Conclusions

In this study, GHSC was prepared from the skin of greenfin horse-faced filefish and identified as typical type I collagen. We confirmed that the morphology, structure, functional, and rheological properties of GHSC are pH dependent. A higher pH caused the GHSC morphology to change from a well-textured ordered stack to a loose silk-like structure. Meanwhile, as the pH was increased, GHSC gradually unfolded, with the α-helices and β-turns decreasing, β-sheets and random coils increasing, and random coils becoming the dominant conformation. The WAC was influenced by morphology and secondary structure, with pH increasing β-sheets and random coils and decreasing α-helices, making GHSC more hydrophobic. A further increase in pH with a rough and silk-like morphology lead to increased hydrophilicity of the GHSC surface. GHSC has the potential to be used as an emulsifier and a foaming agent in pharmaceutical delivery materials and in the food industry when the pH value is far from the pI. In addition, the functional properties were affected by the net surface charge and secondary structure of GHSC, where an increase in the net surface charge of GHSC and the random coil conformation enhanced its emulsification and foaming properties. The pH affects its viscoelasticity, with increasing pH causing the GHSC solution to change from a dominant viscous to elastic behaviour. The viscous behaviour at pH below pI offers GHSC the potential to be used in injectable biomaterials; the elastic behaviour at pH above pI makes GHSC suitable for use as a hydrogel for wound dressings. In conclusion, we investigated the changes in the structure, function and rheological properties of GHSC in the skin of greenfin horse-faced filefish upon pH treatment, which can provide theoretical support and guidance for the application of GHSC in the fields of food, medicine and biomedicine.

## Data Availability

Data are contained within the article.
